# Feasibility of Low Fermentable Oligosaccharide, Disaccharide, Monosaccharide, and Polyol Diet and Its Effects on Quality of Life in an Italian Cohort

**DOI:** 10.3390/nu12030716

**Published:** 2020-03-08

**Authors:** Arianna Cingolani, Danilo Paduano, Valentina Vecchiarelli, Manuela Demelas, Paola Teresa Corrias, Laura Casula, Paolo Usai

**Affiliations:** 1 Gastroenterology, Department of Medical Sciences and Public health, Policlinico Universitario di Monserrato, University of Cagliari, 09042 Monserrato (CA), Italy; danilo.paduano@libero.it (D.P.); valentinavecchiarelli@gmail.com (V.V.); paolousai50@gmail.com (P.U.); 2 Department of Medical Sciences and Public Health, University of Cagliari, 09242 Monserrato (CA), Italy; manu.demelas@tiscali.it (M.D.); paolygin@yahoo.it (P.T.C.); laura.casula@gmail.com (L.C.)

**Keywords:** diet, FODMAPs, feasibility, quality of life

## Abstract

The low Fermentable oligosaccharides, disaccharides, monosaccharides, and polyols (FODMAP) diet has demonstrated excellent results in terms of symptom control and health-related quality of life (HRQoL) in irritable bowel syndrome (IBS) sufferers, but patients have complained about unsatisfying taste, difficulty in following the diet, and time consumption. To investigate the feasibility of the low FODMAP diet in an Italian (Sardinian) cohort, sixty consecutive eligible outpatients (11 men and 49 women) with IBS were enrolled and followed a low FODMAP diet (gluten allowed, restriction phase of four weeks, reintroduction phase of four weeks). Food habits were assessed using 24-hour dietary recall, Bristol Stool Scale for stool consistency, Visual Analogue Scale for abdominal bloating, VAS (Visual Analogue Scale) for abdominal pain, IBS Severity Scoring System for perceived disease severity, and a 12-item Short Form Survey for HRQoL (psychological component summary + mental component summary) were applied at baseline (T0) and at the end of each phase (T1-four weeks and T2-eight weeks). Statistical analysis was performed by dividing the cohort into diarrhoea-dominant IBS (IBS-D) and other IBS subtypes (selected IBS-others). Comparisons between T1 and T2 vs. T0 and T2 vs. T1 were performed. The low FODMAP diet lowered VASp (VAS pain), VASb (VAS bloating), and IBS SSS (IBS Severity Scoring System), and increased PCS (Physical Component Summary) and MCS (Mental Component Summary) in both subgroups. Bristol Stool Scale (BSS) only improved in the IBS-D subgroup. The dropout mean values for MCS were higher than treated subjects and the percentage of unemployment was lower in the dropouts. According to the dropout features, the low FODMAP diet seems to show greater feasibility for patients with more time to dedicate to the diet (unemployed, homemakers, housewives, or students), more motivation, and more severe clinical features, independent of their place of residence.

## 1. Introduction

Irritable bowel syndrome (IBS) is a functional intestinal disorder characterised by chronic or recurrent symptoms which cannot be explained by the presence of structural, metabolic, or biochemical alterations [1]. IBS has a significant effect on the patient’s quality of life [2], both personal and social, and represents an economic burden as a result of increased healthcare costs and absence from work [3,4].

The role of food in the development of functional gastrointestinal disorders (FGIDs)-particularly IBS-has not yet been well-defined. Most traditional dietary recommendations are largely based on expert opinions and common sense rather than on accredited scientific evidence. More recently, with a broader understanding of the pathophysiology of IBS, the potential role of nutrition has been revised [5].

Increasing evidence has indicated that fermentable oligosaccharides, disaccharides, monosaccharides, and polyols (FODMAPs) contribute to the development of symptoms in patients with IBS or other FGIDs, although they are not the cause of IBS [6].

FODMAPs are short-chain carbohydrates which are present in a wide range of foods and are poorly absorbed in the small intestine and highly fermentable by the colonic bacterial flora. More specifically, they include fructose, lactose, fructans (or fruit-oligosaccharides), galacto-oligosaccharides (GOS), and polyols, such as sorbitol and mannitol [7].

A FODMAP-rich diet significantly worsens symptoms (abdominal pain, bloating, and nausea) in patients with IBS compared to healthy volunteers who show mild or no symptoms. It is worth mentioning that these symptoms develop after the first day of following such a diet and have been associated with increased gas production [8].

While pharmacological therapies, such as prucalopride, linaclotide, and lubiprostone, are effective and relatively safe for the IBS-constipation variant [9], in general, diet and food choice are initially considered for patients with IBS in order to improve their gut symptoms [10].

Dietary strategies in IBS management are supported by a wide array of scientific evidence; these are typically elimination diets (e.g., gluten-free), reduced content of fermentable substrates (e.g., low lactose), or elimination/rechallenge diets (e.g., low FODMAP diet) [10].

These dietary strategies commonly improve IBS symptoms, but diverse data assessing the effects and long-term acceptability of elimination diets are relatively few. Additionally, elimination diets are known to be time- and labour-consuming [11].

Specifically, according to the 10-year studies and practices of the low FODMAP diet, this type of diet improves the symptoms and health-related quality of life (HRQoL) in patients with IBS; however, patients often complain of unsatisfying taste and have low acceptance towards the dietary plan, as well as often reporting that this diet is significantly difficult to follow [12].

The Italian and Sardinian population’s traditional diet is a typical Mediterranean one, characterised by high consumption of vegetables, fruits, nuts, legumes, and unprocessed cereals; low consumption of red meat and meat products; moderate or high consumption of fish; and low consumption of yogurt and seasoned cheeses as main dairy products. Alcohol is moderately consumed during meals. The total intake of lipids can be approximately 40% of the total intake, with a high ratio of unsaturated lipids due to the high monounsaturated content of olive oil, which is used as the main culinary fat [13].

Moreover, in Italy, adult-type hypolactasia is significantly common, as in all Mediterranean areas; in fact, in some regions, approximately 90% of the adult population carries the C/C 13910 LNP allele and approximately 9% carry the C/T 13910 allele, both of which are implicated in lactase non-persistence [14].

Nonetheless, food and taste are a direct expression and consecution of local cultures, and every food culture differs from others in terms of FODMAP quality and quantity; thus increasing the complexity of the problem of the applicability and acceptability of the low FODMAP dietary plan [15].

Therefore, more data are required to optimise the choices in IBS therapy and management of symptoms in order to offer a tailored treatment for each patient with IBS.

This study aims to determine which IBS patients could benefit more from FODMAP reduction and to investigate the feasibility and applicability of such a dietary plan in an Italian (Sardinian) cohort with different demographic features.

## 2. Materials and Methods 

**Figure 1:**
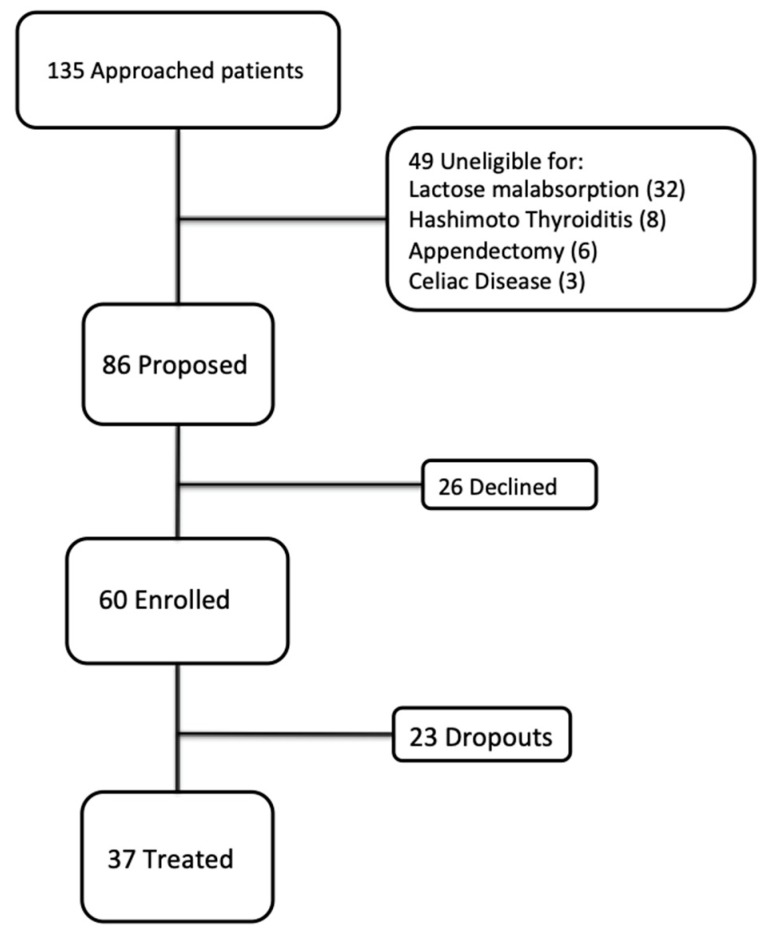
Study flow chart.

We enrolled 60 consecutive eligible patients (11 men and 49 women; mean age 37.26 y) (Figure 1) with IBS (according to the Rome IV criteria) who had been admitted to the Clinic of the Gastroenterology Unit of the University Hospital of Cagliari (Italy) between September 2018 and March 2019. After being extensively informed about the aim and design of the study, the subjects provided written informed consent prior to study inclusion.

The exclusion criteria were as follows: a history of gastrointestinal organic diseases, clinically significant systemic diseases, established food allergies, eating disorders, and major abdominal surgery. Celiac disease, intestinal inflammation, lactose malabsorption, and thyroid diseases were excluded through IgA and IgG antitransglutaminase antibody dosage, faecal calprotectin, lactose hydrogen breath test, and dosage of the thyroid-stimulating hormone, free triiodothyronine, and free thyroxine, respectively.

All enrolled patients were not allowed to take laxatives, antidiarrheal drugs, antimicrobials, or probiotics during the trial.

According to the Rome IV criteria, subjects were classified into the four IBS subtypes: diarrhoea-dominant IBS (IBS-D), constipation-dominant IBS (IBS-C), mixed diarrhoea and constipation (IBS-M), and indeterminate variant (IBS-U).

Data on their eating habits were retrospectively collected in conformity using the 24-hour dietary recall method [16] by a nutritionist.

Perceived health-related quality of life (HRQoL) was studied using a 12-Item Short Form Survey (SF-12) questionnaire [17], which consisted of a Physical Component Summary (PCS) and a Mental Component Summary (MCS), to investigate the perceived disability and limitations regarding both psychological and physical states, both ranging from 0 to 50. Perceived severity of disease was investigated through the IBS Severity Scoring System [18], which ranges from 0 to 500, and the severity of abdominal pain and bloating were investigated through the Visual Analogue Scale [19] (VAS pain and VAS bloating), which ranges from 0 to 10 in an analogical scale. Stool type was evaluated according to the Bristol Stool Scale (BSS) [20], which ranges from 1 (hard stools) to 7 (loose stools), grades 3 and 4 being considered normal. All of these measurements were performed at enrolment (T0), after 4 weeks (elimination phase of the diet, T1), and at the time of the reintroduction phase after 4 more weeks (T2). As for IBS-SSS, a drop of 30% was considered clinically significant and a value <75 was considered remission. For PCS and MCS, an increase of 20% from baseline was considered clinically significant. VAS pain (VASp) and VAS bloating (VASb) decreases of at least 20% were considered significant. As for BSS, the tendency towards the grades 3 and 4 was considered a clinical improvement.

The study was conducted in concordance with the Declaration of Helsinki and approved by the Independent Ethics Committee of the Cagliari University (code PG/2018/8836).

### 2.1. Diet

Patients were advised by a trained nutritionist of a dietary plan to reduce the FODMAPs from their diet over a period of four weeks and gradually reintroduce this type of diet, one favourite kind of FODMAPs at a time, according to a specific dietary management for a further four weeks.

Data were analysed using the “Progetto Dieta” software to obtain the following: the daily calories burned (in kcal); the percentage and weight of daily proteins, carbohydrates, and lipids; the alcohol intake; and the number of daily portions of fruits.

The cohort had an average basal metabolism of 1.350 ± 167 kcal and a daily consumption of 1.994 ± 612 kcal (Table 1). A total of 25% of the patients skipped breakfast and snacks, concentrating all their nutrients into two main meals (lunch and dinner). 

The diet assigned to each patient was elaborated using the “Progeo Nutrigeo” program and referred to the 2014 LARN (Reference Levels of Nutrients and Energy), updated by the SINU (Italian Society of Human Nutrition) and addressed to the Italian population [21].

The macronutrients to be consumed daily were carbohydrates (45%–60%), lipids (20%–35%), and proteins (10%–15%). Balanced diets, by varying and harmonizing the meals, prevent patients from an excessive FODMAP charge, as has been recently observed by our group [12]. Thus, to balance nutrients and redistribute meals throughout the day, the diet was organized into three main meals with two snacks between them. Furthermore, specific recommendations were given in addition to the reduction of FODMAP intake (low FODMAP diet). Patients were advised to pay careful attention to the labels of packaged foods, and to avoid the use of laxatives, prokinetics, or antidiarrheal drugs. 

A diet is considered to be low in FODMAPs if it provides less than 0.5 g by intake or less than 3 g/day (the Australian diet contains, on average, 23.7 g of FODMAPs/day) [22]. Studies or tables regarding the usual intake or food composition in FODMAPs are not currently available in Italy.

The dietary plan we provided, based on the reduction of all FODMAPs (with gluten allowed), was designed using an elimination phase and a reintroduction phase [22].

The exclusion phase had to be maintained for 4 weeks followed by a non-summatory reintroduction of foods removed by groups of different FODMAPs to avoid additive effects and to identify the possible individual intolerances for each group. The long-term patients should be able to control their symptoms by consuming foods that contain FODMAPs, according to their tolerance limits. This dietary model did not include gluten restriction.

A low FODMAP diet restricts the consumption of fructose, fructo-oligosaccharides, GOS, lactose, and polyols. In practice, the avoidance of these components implies the avoidance of the foods listed in Table 2 [22].

In the reintroduction phase, the patients progressively (in a non-summatory manner) reintroduced the foods which were previously removed, by groups. For example, only high fructose foods were reintroduced in the first week, only high lactose foods in the second week (excluding the high fructose foods tested in the first week), and only high fructan foods in the third week (Table 3).

If symptoms recur after the reintroduction of one of the groups, that group should be removed again and the process continued with the next group [22].

### 2.2. Statistical Analysis

Statistical analysis was performed by dividing the population into two subgroups: IBS-D vs. other IBS subtypes (selected IBS-others).

Continuous variables were reported as mean ± SD and categorical variables as the number of cases and percentage. The Mann–Whitney U test was used to assess the existence of any differences between the quantitative variables under examination at T0 between the two groups. Paired t-test was used to evaluate the difference in the mean values of the quantitative variables between T0, T1, and T2 and between T1 and T2. A P-value level of <0.05 was considered significant for all tests, and Bonferroni correction for multiple tests was applied; in temporal comparisons, we considered a critical value of 0.0167.

Analyses were performed using the Stata/SE version 14 software (StataCorp LP, College Station, TX, United States).

## 3. Results

Of the 60 patients enrolled, 37 (eight men and 29 women) underwent follow-ups at four and eight weeks since their first visit; 23 (three men and 20 women) did not undergo follow up and were classified as dropouts. Of the 37 patients who underwent the entire protocol, according to the Rome IV criteria, 18 (48.6%) had an IBS-D subtype, 12 (32.4%) IBS-M subtype, 4 (10.8%) IBS-C subtype, and 3 (8.2%) IBS-U subtype.

### 3.1. Mental Component Summary (MCS) and Psychological Component Summary (PCS)

Mental Component Summary and Psychological Component Summary were used to investigate the perceived health-related quality of life.

At the time of their first visit (T0), the IBS-D subjects had a mean MCS score of 34.86 ± 9.81 and a mean PCS score of 39.12 ± 8.9, while the IBS-other subgroups had a mean MCS score of 37.01 ± 13.68 and a mean PCS score of 41.24 ± 10.01. Compared to T0, at T1, a clearer improvement in MCS and PCS in both IBS-D (*p* < 0.0000 and *p* = 0.0001, respectively) and IBS-others (*p* = 0.0008 and *p* = 0.0006, respectively) was observable.

At T2, an improvement in MCS and PCS was more visible compared to T0: both in IBS-D (MCS: *p* < 0.000, PCS: *p* < 0.000) and IBS others (*p* = 0.0003 and *p* = 0.0002, respectively). In T2 vs. T1, the improvement was slight: *p* = 0.0053 and *p* = 0.0441 for MCS and PCS in IBS-D and *p* = 0.0493 in MCS for IBS others. Improvement in PCS was not observed in IBS-others for T2 vs. T1. (Table 4).

### 3.2. Irritable Bowel Syndrome Severity Scoring System

At T0, an average score of 313.33 ± 74.99 for IBS-D and 320.53 ± 79.27 for IBS-others was observed; these values were indicative of severe intensity. A considerable difference was observed when comparing T1 vs. T0 (*p* < 0.0000 for both IBS-D and IBS-others) and T2 vs. T0 (*p* < 0.0000 for both IBS-D and IBS-others), reaching the mean scores indicative of mild intensity. The difference between T2 and T1 was not significant (Table 4).

### 3.3. Pain Severity (Visual Analogue Scale [VAS] for Pain)

At T0, an average score of 7.50 ± 1.98 for IBS-D and 7.95 ± 1.39 for IBS-others was observed, indicative of severe intensity. A considerable difference was observed when comparing T1 vs. T0 (*p* < 0.0000 for both IBS-D and IBS-others) and T2 vs. T0 (*p* < 0.0000 for both IBS-D and IBS-others), reaching mean scores indicative of mild intensity. Significant differences when comparing T2 vs. T1 were not observed (Table 4).

### 3.4. Bloating Severity (VAS Bloating)

At T0, average scores of 7.17 ± 1.79 for IBS-D and 7.79 ± 1.65 for IBS-others were observed, indicative of severe intensity. A significant decrease was observed when comparing T1 vs. T0 (*p* < 0.0000 for both IBS-D and IBS-others) and T2 vs. T0 (*p* < 0.0000 for both IBS-D and IBS-others), reaching mean scores indicative of mild intensity. The improvement was not significant when comparing T2 vs. T1 (Table 4).

### 3.5. Stool Consistency (Bristol Stool Scale)

At baseline, the mean BSS scores were 6.28 ± 1.45 for IBS-D and 3.95 ± 2.91 for IBS-others (*p* = 0.0457, Mann–Whitney U test); values between 3 and 5 or a tendency towards these values are considered normal, while values of 1–2 or 6–7 are considered pathologic. There was a significant improvement in BSS between T1 vs. T0 (p < 0.0000) and T2 vs. T0 (*p* < 0.0000), but only for the IBS-D subgroup. The improvement was not significant for IBS-other subgroups, when comparing T2 and T1 both for IBS-D and IBS-others (Table 4)

### 3.6. Dropout Analysis

#### 3.6.1. PCS Analysis and MCS Dropouts vs. Treated Patients

The dropout mean values for PCS and MCS variables were significantly higher (46.6 and 42.1) compared to the mean PCS and MCS values of the patients who performed both follow-ups (40.2 and 36.0; MCS *p* = 0.003; PCS *p* = 0.073).

#### 3.6.2. Analysis of the Social Activities of Dropouts and Treated Patients

Analysis of the specific item for the social activities at T0 of the 37 treated patients and 23 dropouts showed that the answers “always,” “almost always,” and “a part of time” were reported by 54.05% of the treated patients and by 47.60% of the dropouts, while the answers “never” and “hardly ever” were given by 45.94% of treated patients and 52.40% of dropouts.

#### 3.6.3. Differences in Demographic Characteristics between Dropouts and Treated Patients

A total of 65.2% of the dropouts resided at a distance of less than 20 km from the hospital. The percentage of unemployed patients was lower in dropouts (13.0%) than that in treated subjects (35.2%).

#### 3.6.4. Evaluation of Bristol Stool Scale, VAS Pain, and VAS Bloating in Dropouts

In the dropouts (23 patients), the average values related to the VAS pain and VAS bloating were significantly lower (3.52 cm and 4.34 cm, respectively) than those in treated patients (7.95 cm and 7.79 cm, respectively; *p* < 0.01). According to the BSS, 52% (12 patients) at T0 had faeces with characteristics ranging from 3 to 5 vs. 6% of the treated patients.

## 4. Discussion

The low FODMAP diet has confirmed its efficacy in the improvement of symptoms and QoL, with stability of all improvements towards the controlled reintroduction phase. The maintenance of changes between T1 and T2 could be attributed to the fact that patients, during the controlled reintroduction phase, learned to identify their tolerance for each FODMAP group and to control their symptoms by avoiding what they cannot tolerate.

Therefore, modifying the diet by reducing the daily FODMAP intake, with subsequent reduction of osmotic activity, intestinal secretion, sensitivity, and motility exerted by such carbohydrates in the intestinal lumen, can lead to a significant improvement in the health-related quality of life, with recovery of an acceptable social, emotional, and working life in our patients. These data confirm what has recently been described in several studies [23]. This was noted particularly in IBS-D patients, probably due to the reduction of osmotic action exerted by FODMAPs in the intestinal lumen. 

After four and eight weeks, the diet was able to significantly increase the stool consistency in IBS-D patients. 

Besides the modification of stool consistency and intestinal movement, we also saw a significant improvement in both VAS pain and VAS bloating, with a decrease in the IBS Severity Score in all IBS types. This could be secondary to the reduction of gas production and by the improvement of intestinal motility, as previously noted by Ong et al. [8].

These improvements could also be attributed to the fact that we provided for the redistribution of nutrients and FODMAPs in five meals in order to avoid concentrating the supply of fermentable sugars over a limited space of time. 

The following are the well-known disadvantages of a low-FODMAP diet: it is complex, difficult to teach to the subjects, difficult to follow up on, and labour intensive. The first dietary management counselling appointment is typically estimated to last approximately one hour. At this time, the required commitment has led to some reluctance for physicians to recommend this diet to patients. Incomplete education may subsequently lead to partial or complete non-compliance in clinical practice. Therefore, nutritional counselling over time is essential for adherence, success, and therapeutic consolidation.

Employment status seemed to affect adherence to the study, as demonstrated by the fact that the percentage of unemployed patients was lower in dropout patients (13%) than in the treated subjects (35.2%). Working conditions are an important social determinant of health; this could be the reason for the higher scores of PCS and MCS in dropouts. These results were consistent with the findings reported in several studies in which the level of education and employment has been associated with higher QoL scores [24].

Consequently, probably because of the limited availability of time due to their working activity, poor motivation, better health conditions, and shorter available time to devote to medical checks, adherence to visits and medical prescriptions was lower in the employed patients.

Additionally, patients need to devote their time to planning and purchasing foods for a low FODMAP diet, which can also reduce compliance. This aspect may be improved by providing detailed tables on the FODMAP content of foods, which are specifically addressed to each population. Such tables already exist in some countries.

Regarding the dropouts, from a demographic analysis it was possible to exclude the distance from the hospital as a possible cause of study dropout. In contrast, employment status, education, and the impact of the disease on social relationships were less evident.

Adequate information and diet education provided by a nutritionist or a physician experienced in functional GI disorders in an appropriate time is a fundamental factor in a patient attaining a full awareness and compliance to the strategy of the dietary plan. 

Unfortunately, this work presents several weaknesses, including the following: the limited number of patients studied, the high number of dropouts, the objective difficulty of having reliable information regarding patient’s adherence to the diet as outpatients, and the lack of criteria to establish whether the patient had followed the suggested food advice; both in regards to the consumed food and quantifying the daily consumption of FODMAPs.

We have not eliminated the placebo effect of the diet; in fact, the diet was offered to patients without randomisation vs. placebo, and no blinding of healthcare professionals and patients was performed. Moreover, the results were based on the patient’s reports, which could have been influenced by the fact they knew what the diet provided.

The study population, as in the majority of IBS studies, was unbalanced towards the female sex; this could have led to bias, but it is a direct representation of the demographic characteristics of the IBS patients who were referred to our department. Moreover, patient enrolment was even more challenging due to the high prevalence of lactose malabsorption in Sardinia, limiting the number of eligible patients even more.

## 5. Conclusions

In this study, the low FODMAP diet has confirmed its efficacy in improving IBS symptoms such as abdominal pain, bloating, stool consistency, perceived severity of disease, and physical and mental components of quality of life; particularly in IBS-D patients. The low-FODMAP diet should be associated with food re-education in order to enable patients to make a conscious choice about various foods.

This dietary plan may be more feasible for patients with more dedication to the diet (students, unemployed, homemakers, housewives) and more motivation (more severe clinical features), in contrast to patients with full-time occupations (clerks, workmen, freelancers) or mild symptoms, independent of their place of residence and distance from the hospital.

Our study presents some weaknesses, such as the absence of randomisation, possible placebo effect of the diet, and the possibility in Italy to conduct such studies only in outpatients—which are mostly women—in a population with a high prevalence of lactose malabsorption. Additionally, there was no chance to measure the exact content of FODMAPs of each home-made meal, but only to estimate it due to both the absence of detailed data and no possibility of providing meals to participants. These aspects need to be improved in future research.

## Figures and Tables

**Table 1:** Mean nutrients and energy intake of the cohort at baseline.

Variables	Mean ± SD	Percent of Daily Intake
Proteins (g)	76 ± 25	17 ± 4 (%)
Lipids (g)	65 ± 24	28 ± 9 (%)
Carbohydrates (g)	258 ± 120	54 ± 12 (%)
Alcohol		0.05 ± 0.01 (%)
Fruit and vegetable portions (number of)	2.5 ± 1.3	
Basal metabolism (kcal)	1350 ± 167	
Daily consumption (kcal)	1994 ± 612	

**Table 2:** Food with high fermentable oligosaccharides, disaccharides, monosaccharides, and polyol (FODMAP) content divided by groups (reintroduction phase). Adapted from reference [22].

**Fructose group**	Fruit: apple, pear, cantaloupe, Nashi pear, peach, mango, grapes, cherry, watermelon, canned fruit in natural juice sweeteners: honey, fructose, high fructose corn syrup, ‘light’ jams with fructose Vegetables: asparagus, artichoke, green peas High fructose doses: concentrated fruit sources; large servings of fruit, dried fruits, fruit juices
**Lactose group**	Milk: cow, goat, and sheep; ice cream Yoghurt, custard, junket Cheese: soft and fresh (e.g., ricotta, cottage, mascarpone)
**Fructan group**	Cereals: wheat, rye, barley, when taken in large amounts (e.g., bread, pasta, couscous, crackers, biscuits) Onion, shallot, garlic. Vegetables: artichoke, asparagus, beet, Brussels sprouts, cabbage, broccoli, fennel, leek, green peas, chicory. Fruit: khaki, watermelon. Nuts: walnut, hazelnut, pistachio. Inulin (laxatives, enteral nutrition, soya drink)
**Galactan** **group**	Pulses: Beans, chickpeas, lentils, soybeans
**Polyol group**	Fruit: apple, apricot, pear, nectarine, watermelon, custard apple, peach, khaki, lychee, cherry, prune, raisins, cantaloupe, blackberry, avocado. Vegetables: cauliflower, mushrooms, green peas, green pepper. Sweeteners: sorbitol (E420), mannitol (E421), xylitol (E967), maltitol (E965), isomaltitol (E953), and others ending in ‘-ol’

**Table 3:** Score values: Visual Analogue Scale (VAS) bloating and VAS pain (0–10); Bristol stool chart (1–7); Irritable Bowel Syndrome Severity Scoring System (0–500); physical component summary and mental component summary side (0–50).

VARIABLE (T0)	IBS_Others (Mean ± SD)	IBS_D (Mean ± SD)	*p*-Value *
VAS pain	7.95 ± 1.39	7.50 ± 1.98	0.4648
VAS bloat.	7.79 ± 1.65	7.17 ± 1.79	0.2775
BSS	3.95 ± 2.91	6,28 ± 1.45	0.0457
IBS SSS	320.53 ± 79.27	313.33 ± 74.99	0.9514
PCS	41.24 ± 10.01	39.12 ± 8.90	0.6054
MCS	37.01 ± 13.68	34.86 ± 9.81	0.6816

* Mann–Whitney U test.

**Table 4:** Difference in the mean values of scores between T0, T1, and T2. Score values: Visual Analogue Scale (VAS) bloating and VAS pain (0–10); Bristol stool chart (1–7); Irritable Bowel Syndrome Severity Scoring System (0–500); physical component summary and mental component summary side (0–50).

	VARIABLE (T_1_ vs. T_0_)	IBS_Others (Mean ± SD)	Test *	IBS_D (Mean ± SD)	Test *
**T1 T1 vs. T0**	VAS pain	−5.11 ± 2.81	0.0000	−5.72 ± 2.74	0.0000
	VAS bloat.	−5.74 ± 2.35	0.0000	−4.72 ± 2.91	0.0000
	BSS	−0.53 ± 2.97	0.4498	−2.72 ± 0.89	0.0000
	IBS SSS	−169.47 ± 76.12	0.0000	−165.56 ± 66.71	0.0000
	PCS	8.66 ± 9.12	0.0006	9.42 ± 7.92	0.0001
	MCS	13.84 ± 14.99	0.0008	16.66 ± 10.01	0.0000
**T2 vs. T0**	VAS pain	−5.05 ± 2.63	0.0000	−5.72 ± 2.65	0.0000
	VAS bloat.	−5.68± 2.19	0.0000	−4.89 ± 2.56	0.0000
	BSS	0.53 ± 3.01	0.4554	−2.50 ± 1.10	0.0000
	IBS SSS	−183.16 ± 69.94	0.0000	−167.50 ± 71.77	0.0000
	PCS	9.92 ± 9.13	0.0002	11.76 ± 9.08	0.0000
	MCS	15.27 ± 14.72	0.0003	19.20 ± 10.42	0.0000
**T2 vs. T1**	VAS pain	0.05 ± 1.08	0.8340	0.00 ± 0.34	10.000
	VAS bloat.	0.05 ± 1.13	0.8413	−0.17 ± 0.86	0.4210
	BSS	0.00 ± 1.00	10.000	0.22 ± 0.73	0.2151
	IBS SSS	−13.68 ± 36.81	0.1225	−1.94 ± 23.65	0.7315
	PCS	1.25 ± 5.16	0.3036	2.33 ± 4.55	0.0441
	MCS	1.43 ± 2.96	0.0493	2.54 ± 3.37	0.0053

* Paired t - test.

## References

[1] Longstreth G.F. (2005). Definition and Classification of Irritable Bowel Syndrome: Current Consensus and Controversies. Gastroenterol. Clin. North Am..

[2] Hou X., Chen S., Zhang Y., Sha W., Yu X., ElSawah H., Afifi A.F., El Khayat H., Nouh M.A., Hassan M.F. (2014). Quality of life in patients with Irritable Bowel Syndrome (IBS), assessed using the IBS-Quality of Life (IBS-QOL) measure after 4 and 8 weeks of treatment with mebeverine hydrochloride or pinaverium bromide: Results of an international prospective observational cohort study in Poland, Egypt, Mexico and China. Clin. Drug Investig..

[3] Buono J.L., Mathur K., Averitt A.J., Andrae D.A. (2017). Economic Burden of Irritable Bowel Syndrome with Diarrhea: Retrospective Analysis of a U.S. Commercially Insured Population. J. Manag. Care Spéc. Pharm..

[4] Doshi J.A., Cai Q., Buono J.L., Spalding W.M., Sarocco P., Tan H., Stephenson J.J., Carson R.T. (2014). Economic Burden of Irritable Bowel Syndrome with Constipation: A Retrospective Analysis of Health Care Costs in a Commercially Insured Population. J. Manag. Care Pharm..

[5] Barrett J.S., Gibson P.R. (2012). Fermentable oligosaccharides, disaccharides, monosaccharides and polyols (FODMAPs) and nonallergic food intolerance: FODMAPs or food chemicals?. Ther. Adv. Gastroenterol..

[6] Barrett J.S., Gearry R., Muir J.G., Irving P.M., Rose R., Rosella O., Haines M.L., Shepherd S., Gibson P.R. (2010). Dietary poorly absorbed, short-chain carbohydrates increase delivery of water and fermentable substrates to the proximal colon. Aliment. Pharmacol. Ther..

[7] Spiller R.C. (2017). How do FODMAPs work?. J. Gastroenterol. Hepatol..

[8] Ong D.K., Mitchell S.B., Barrett J.S., Shepherd S.J., Irving P.M., Biesiekierski J., Smith S., Gibson P., Muir J.G. (2010). Manipulation of dietary short chain carbohydrates alters the pattern of gas production and genesis of symptoms in irritable bowel syndrome. J. Gastroenterol. Hepatol..

[9] Thayalasekeran S., Ali H., Tsai H.H. (2013). Novel therapies for constipation. World J. Gastroenterol..

[10] Gibson P., Varney J., Malakar S., Muir J.G. (2015). Food Components and Irritable Bowel Syndrome. Gastroenterology.

[11] McKenzie Y.A., Alder A., Anderson W., Wills A., Goddard L., Gulia P., Jankovich E., Mutch P., Reeves L.B., Singer A. (2012). British Dietetic Association evidence-based guidelines for the dietary management of irritable bowel syndrome in adults. J. Hum. Nutr. Diet..

[12] Paduano D., Cingolani A., Tanda E., Usai P. (2019). Effect of Three Diets (Low-FODMAP, Gluten-free and Balanced) on Irritable Bowel Syndrome Symptoms and Health-Related Quality of Life. Nutrients.

[13] Gonzalez M.A.M., Martín-Calvo N. (2016). Mediterranean diet and life expectancy; beyond olive oil, fruits, and vegetables. Curr. Opin. Clin. Nutr. Metab. Care.

[14] Obinu D.A., Enattah N.S., Pedroni A., Peltonen L., Cavalli-Sforza L.L., Dore M.P. (2010). Prevalence of lactase persistence and the performance of a non-invasive genetic test in adult Sardinian patients. e-SPEN, Eur. e-J. Clin. Nutr. Metab..

[15] Hewawasam S.P., Iacovou M., Muir J.G., Gibson P. (2018). Dietary practices and FODMAPs in South Asia: Applicability of the low FODMAP diet to patients with irritable bowel syndrome. J. Gastroenterol. Hepatol..

[16] Castell G.S., Serra-Majem L., Ribas-Barba L. (2015). What and how much do we eat? 24-hour dietary recall method. Nutr. Hosp..

[17] Ware J., Kosinski M., Keller S.D. (1996). A 12-Item Short-Form Health Survey: Construction of scales and preliminary tests of reliability and validity. Med. Care.

[18] Pedersen N., Vegh Z., Burisch J., Jensen L., Ankersen D., Felding M., Andersen N.N., Munkholm P. (2014). Ehealth monitoring in irritable bowel syndrome patients treated with low fermentable oligo-, di-, mono-saccharides and polyols diet. World J. Gastroenterol..

[19] Bengtsson M., Ohlsson B., Ulander K. (2007). Development and psychometric testing of the Visual Analogue Scale for Irritable Bowel Syndrome (VAS-IBS). BMC Gastroenterol..

[20] Blake M., Raker J.M., Whelan K. (2016). Validity and reliability of the Bristol Stool Form Scale in healthy adults and patients with diarrhoea-predominant irritable bowel syndrome. Aliment. Pharmacol. Ther..

[21] (2014). Livelli di Assunzione di Riferimento di Nutrienti ed Energia per la Popolazione Italiana.

[22] Murillo A.Z., Arévalo F.E., Jáuregui E.P. (2016). Diet low in fermentable oligosaccharides, disaccharides, monosaccharides and polyols (FODMAPs) in the treatment of irritable bowel syndrome: Indications and design. Endocrinol. y Nutr..

[23] Yepes I.D.J., Múnera M.N., Martelo C. (2017). Diet low in fermentable oligosaccharides, disaccharides, monosaccharides and polyols, and quality of life in patients with irritable bowel syndrome in Colombia. Biomédica.

[24] Patti F., Pozzilli C., Montanari E., Pappalardo A., Piazza L., Levi A., Onesti E., Pesci I., The Italian Study Group on Quality of Life in MS (2007). Effects of education level and employment status on HRQoL in early relapsing-remitting multiple sclerosis. Mult. Scler. J..

